# Patient Blood Management for a Transfusion Dilemma Associated With a Rare Blood Group: A Case Report

**DOI:** 10.7759/cureus.113560

**Published:** 2026-07-28

**Authors:** Zakaa Majeed, Farah Abu Kahila, Ishma Aijazi, Nisha Vasudevan Navin, Mouza Abdullah AlSharhan, Jamila Mohammed Bin Adi, Hiba Alhumaidan

**Affiliations:** 1 Department of Laboratory Medicine and Pathology, Dubai Health, Dubai, ARE; 2 Department of Internal Medicine, Dubai Health, Dubai, ARE

**Keywords:** anti‑inᵇ alloantibody, indian blood group system, iron deficiency anemia, patient blood management, severe anemia

## Abstract

Patient blood management (PBM) is a patient-centered, evidence-based approach aimed at improving outcomes by optimizing and preserving a patient’s own blood, particularly when transfusion support is limited.

We report a 76-year-old female with congestive heart failure and severe mitral stenosis presenting with severe iron deficiency anemia. Transfusion was not feasible due to an alloantibody against a high-frequency antigen (HFA), later identified as anti‑Inᵇ in a rare In(b−) phenotype. The patient was managed conservatively using PBM principles, including intravenous iron therapy, minimization of blood loss, and optimization of physiological tolerance to anemia through infection control. Hemoglobin levels improved steadily, and the patient remained stable without requiring a transfusion.

This case highlights the importance of transfusion services and multidisciplinary collaboration in safely managing severe anemia in patients with rare blood groups/antibodies to HFAs.

## Introduction

Severe anemia in elderly patients with underlying cardiovascular disease represents a major clinical challenge, as diminished oxygen-carrying capacity may precipitate myocardial ischemia and heart failure decompensation. Although blood transfusion is commonly employed in the management of symptomatic anemia, contemporary practice emphasizes individualized decision-making based on clinical status rather than hemoglobin thresholds [1,2]. In certain scenarios, however, transfusion may be limited or not feasible, particularly in patients with rare blood groups or clinically significant alloantibodies directed against high-frequency antigens (HFAs), which restrict the availability of compatible donor units [3,4].

The Indian blood group system comprises six antigens, among which the Inᵇ antigen is highly prevalent across most populations. Individuals lacking the Inᵇ antigen (Inb−) are therefore exceedingly rare, and the development of anti‑Inᵇ alloantibodies poses a significant transfusion challenge due to near-universal donor incompatibility [5-8]. The identification of such antibodies requires advanced immunohematological investigations, often involving reference laboratories, and may result in substantial delays or inability to provide compatible transfusion support in both acute and elective settings [4,9].

Patient blood management (PBM) has emerged as an evidence-based, patient-centered strategy aimed at optimizing erythropoiesis, minimizing blood loss, and enhancing physiological tolerance to anemia, thereby reducing reliance on allogeneic transfusion [10]. This approach is particularly valuable in clinical situations where transfusion is constrained by compatibility issues, limited availability of matched blood, or heightened risk of adverse reactions [3,10]. Effective implementation of PBM requires a coordinated multidisciplinary approach involving transfusion medicine specialists, clinicians, and laboratory services.

Despite increasing recognition of PBM, its application in patients with rare blood group phenotypes and complex alloimmunization remains incompletely described in the literature, particularly in the setting of significant cardiovascular comorbidity. We report a case of severe iron deficiency anemia in an elderly woman with underlying cardiac disease and a rare In(b−) phenotype, in whom transfusion was not feasible due to the presence of anti‑Inᵇ alloantibody. This case highlights the successful application of PBM principles as an alternative management strategy and underscores the critical role of interdisciplinary collaboration in navigating complex transfusion scenarios.

## Case presentation

A 76-year-old woman with severe mitral stenosis, congestive heart failure, pulmonary hypertension, and chronic atrial fibrillation on anticoagulation presented with dyspnea, fatigue, and a history of passage of black stools. She had a strong family history of ischemic stroke. On examination, she was hemodynamically stable. Per rectal exam did not reveal melena. 

Investigations

Initial laboratory investigations demonstrated the following important findings (Table 1).

**Table 1:** Initial lab results

Parameter	Result	Lab reference range
Hemoglobin	6.2 g/dL	12.0 - 15.0 g/dL
Serum iron	15 ug/dL	37 - 145 ug/dL
Iron saturation	4%	15 - 45 %
C-reactive protein	10 mg /L	<5.0 mg/L
Prothrombin time	17.6 Sec	12.6 - 16.3 Secs
Activated partial thromboplastin time	43.2 Sec.	25.1 - 37.7 Secs

Blood bank workup 

Based on the mentioned lab results, two RBC units were ordered, and below are the blood bank workup results and interpretation (Table 2), along with the antibody identification panel (Table 3) and reference lab report interpretation:

**Table 2:** Blood bank workup RBC: red blood cell; DTT: dithiothreitol; IBGRL: International Blood Group Reference Laboratory; IAT: indirect antiglobulin test DOa, DOb, COa, and Yta, JMH, are blood group systems

Blood bank test	Result interpretation	
Blood group	A positive	
Antibody screen	Positive, pan-reactive	
Antibody identification	Positive with all panel cells	
Antibody screen (tube)	Room temperature- negative 37^0^C- positive (1+) anti-human globulin phase- 3+	
Auto-control	Negative	
Direct antiglobulin test	Negative (Ruling out the effect of an autoantibody)	
Serological crossmatch	No compatible blood was found (Total of 90 donor units were cross-matched)	
DTT (Dithiothreitol) and Papain enzyme-treated IAT performed	Negative (excluding antibodies against DTT (Dithiothreitol)and enzyme-sensitive antigens). Antibodies against antigens resistant to DTT and enzymes (In and JMH) were not excluded	
Red cell genotyping to rule out/narrow the alloantibody probabilities	Red cell genotyping demonstrated the presence of DOa, DOb, COa, and Yta antigens, thereby excluding corresponding alloantibodies. In and JMH were not tested and hence not ruled out.	
Blood samples were sent out to the International Blood Group Reference Lab (IBGRL)-Bristol-UK extended immunohematology testing and rare blood group identification	Rare phenotype confirmed: In(b−) with anti In(b) antibody

**Table 3:** Antibody identification panel

	RH						Kell		Duffy		Kidd		Lewis		MNS				p	Lu		Result
Reagent RBC	D	C	E	c	e	Cw	K	k	Fya	Fyb	Jka	Jkb	Lea	Leb	M	N	S	s	P1	Lua	Lub	IAT
1	+	+	0	0	+	+	0	+	+	0	+	0	0	0	+	+	+	+	0	0	+	3+
2	+	+	0	0	+	0	+	+	+	0	+	+	0	+	+	0	+	0	+	0	+	3+
3	+	0	+	+	0	0	0	+	+	+	+	0	+	0	+	+	0	+	+	0	+	3+
4	+	0	0	+	+	0	0	+	0	0	+	+	0	0	+	+	+	+	+	0	+	3+
5	0	+	0	+	+	0	0	+	+	+	+	+	0	+	+	0	+	+	+	0	+	3+
6	0	0	+	+	+	0	+	+	0	+	0	+	0	+	0	+	0	+	+	0	+	3+
7	0	0	0	+	+	0	+	+	0	+	+	+	0	+	+	+	+	+	0	0	+	3+
8	0	0	0	+	+	0	0	+	+	+	0	+	+	0	+	+	+	0	+	+	+	3+
9	0	0	0	+	+	0	0	+	+	+	0	+	0	+	0	+	0	+	+	+	+	3+
10	0	0	0	+	+	0	0	+	+	0	+	0	+	+	+	0	+	+	+	+	+	3+
11	+	+	0	0	+	0	0	+	0	+	0	+	+	+	+	0	+	+	0	0	+	3+
Patient cells																						0

Blood samples were referred to the International Blood Group Reference Laboratory (IBGRL), Bristol, United Kingdom, for advanced immuno-hematological investigation. Reference laboratory testing confirmed that the patient possessed the rare In(b−) phenotype and had developed anti‑Inᵇ alloantibody directed against the high-frequency Inᵇ antigen. These findings explained the pan-reactive antibody screen, universal crossmatch incompatibility, and inability to identify compatible donor red blood cell units.

The serological findings of pan-reactivity with a negative auto control, combined with crossmatch incompatibility and reference laboratory confirmation, were consistent with an alloantibody directed against a HFA, specifically anti‑Inᵇ.

Management

A multidisciplinary team involving transfusion medicine, internal medicine, hematology, and cardiology implemented a PBM-based approach due to difficulty in getting compatible blood and the anticipated delay in management.

Management was guided by the three pillars of PBM. Optimization of erythropoiesis (Pillar 1): intravenous iron therapy was initiated to correct iron deficiency and promote red cell production; minimization of blood loss (Pillar 2): anticoagulation was temporarily withheld due to bleeding risk, and blood sampling was restricted; and improving tolerance to anemia (Pillar 3): a urinary tract infection was treated with antibiotics to reduce metabolic demand.

**Figure 1:**
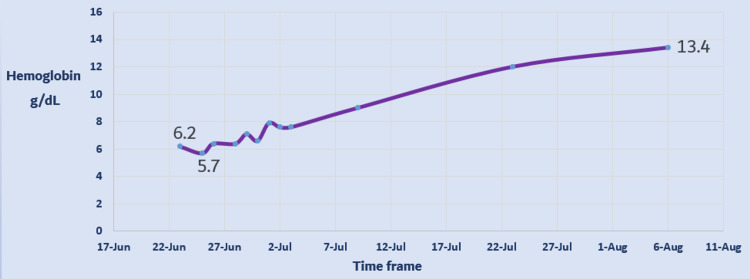
Hemoglobin trend

Endoscopic investigation was initially deferred, but later capsule endoscopy revealed no bleeding source. Hemoglobin improved progressively over three weeks. Anticoagulation (warfarin) was cautiously restarted with close monitoring (Figure 1).

Outcome and follow-up

The patient remained hemodynamically stable with no evidence of ongoing bleeding. Hemoglobin improved steadily from 6.2 g/dl to 9 g/dl in 17 days and to 12 g/dl in four weeks without transfusion. At follow-up, she remained clinically stable on anticoagulation with no further bleeding episodes.

## Discussion

Management of severe anemia in elderly patients with significant cardiovascular comorbidities remains challenging because reduced physiological reserve limits tolerance to impaired oxygen delivery, and red blood cell transfusion is often considered necessary [1,2,11,12]. However, transfusion is not always feasible, particularly in patients with rare blood group phenotypes or alloantibodies directed against HFAs, where compatible blood may be unavailable despite extensive serologic investigation [3,4,9].

Anti‑Inᵇ is an exceptionally rare but clinically significant alloantibody due to the near-universal expression of the corresponding antigen (Inᵇ) in donor populations [3,5-7]. This is supported by large-scale donor screening data from India. Joshi et al. reported that no In(b−) phenotype was identified among 411 serologically tested donors, while molecular screening of 5,261 individuals similarly failed to detect the IN*02.-05 (c.449G>A) variant allele [8]. In a separate study by the same group, a larger cohort of 6,300 donors underwent combined serologic and molecular screening, which likewise did not identify any In(b−) individuals. The authors reported an estimated prevalence of approximately 0.02% based on statistical analysis of the dataset [6]. Collectively, these reports consistently demonstrate the extreme rarity of antigen-negative donors in the studied population. Given the high-prevalence nature of the Inᵇ antigen, compatible donor units are expected to be exceedingly rare, and transfusion support for such patients may require reliance on rare donor registries or international blood bank networks [4,9]. In routine clinical settings, such resources may not be readily available, resulting in substantial delays or an inability to provide transfusion support. Reports describing patients with anti-Inᵇ remain limited, making the present case noteworthy because severe iron deficiency anemia in a patient with significant cardiovascular disease was successfully managed without transfusion despite the absence of compatible blood.

PBM provides an effective evidence-based alternative in such scenarios by optimizing erythropoiesis, minimizing iatrogenic blood loss, and managing comorbid conditions, thereby reducing reliance on allogeneic transfusion [10]. In the present case, multidisciplinary collaboration between internal medicine, hematology, cardiology, and transfusion medicine enabled the successful correction of severe iron deficiency anemia using intravenous iron therapy while avoiding transfusion-related risks.

Importantly, despite marked anemia and underlying valvular heart disease, the patient remained hemodynamically stable throughout treatment. This highlights that transfusion decisions should be guided by the overall clinical condition rather than hemoglobin concentration alone, and that carefully selected patients may safely tolerate lower hemoglobin levels when supported by appropriate monitoring and optimization strategies [1,2,11].

This case further emphasizes the importance of early recognition of complex alloantibody profiles, prompt involvement of transfusion medicine specialists, and timely implementation of PBM in patients with limited transfusion options [3,4,9]. It also underscores the value of multidisciplinary collaboration in achieving favorable outcomes in highly complex transfusion scenarios.

## Conclusions

This case demonstrates that transfusion may not be feasible in patients with rare blood groups and significant alloantibodies. PBM offers a safe and effective alternative when applied through a multidisciplinary approach. Early identification of transfusion limitations and timely PBM implementation are critical to achieving favorable outcomes.

## References

[1] Carson JL, Guyatt G, Heddle NM (2016). Clinical practice guidelines from the AABB: red blood cell transfusion thresholds and storage. JAMA.

[2] Natanson C, Applefeld WN, Klein HG (2024). Hemoglobin-based transfusion strategies for cardiovascular and other diseases: restrictive, liberal, or neither?. Blood.

[3] Tormey CA, Hendrickson JE (2019). Transfusion-related red blood cell alloantibodies: induction and consequences. Blood.

[4] Paccapelo C (2018). Managing a rare donor programme: the immunohaematology laboratory perspective. ISBT Sci Ser.

[5] Abdelmonem M, Ngo N, Cai W (2023). Racial disparity in antibody against a rare high prevalence antigen (anti‑Inᵇ). Am J Clin Pathol.

[6] Joshi SR, Senjaliya SB, Maru HD, Kshirsagar PD, Kulkarni SS, Shrivastava P (2021). A unique approach to screen for blood donors lacking high-prevalence antigen In(b) of the Indian blood group system. Immunohematology.

[7] Lopez GH, Mcbean RS, Wilson B, Irwin DL, Liew YW, Hyland CA, Flower RL (2015). Molecular typing for the Indian blood group associated 252G&gt;C single nucleotide polymorphism in a selected cohort of Australian blood donors. Blood Transfus.

[8] Joshi SR, Senjaliya SB, Srivastava K, Flegel WA (2020). A resource-conserving serologic and highthroughput molecular approach to screen for blood donors with an IN:-5 phenotype. Immunohematology.

[9] Collodel L, Coluccia E, Guaita S, Pivetta M, Vaccara I, Gessoni G (2025). Rare blood group bank in transfusion therapy of patients with complex allo-immunizations. Hemato.

[10] ISBT Working Party (2026). Red cell transfusion thresholds and single-unit transfusion in patient blood management. ISBT.

[11] Coz Yataco AO, Soghier I, Hébert PC (2025). Red blood cell transfusion in critically ill adults: an American College of Chest Physicians Clinical Practice Guideline. Chest.

[12] Goodnough LT, Schrier SL (2014). Evaluation and management of anemia in the elderly. Am J Hematol.

